# Non-arteritic anterior ischemic optic neuropathy – Case report

**Published:** 2018

**Authors:** Monica Bordas, Bogdana Tabacaru, Tudor Horia Stanca

**Affiliations:** * “Prof. Dr. Agrippa Ionescu” Clinical Emergency Hospital, Bucharest, Romania; ** “Carol Davila” University of Medicine and Pharmacy, Bucharest, Romania

**Keywords:** Papillary Edema, Optic Disc Edema, Non-Arteritic Ischemic Optic Neuropathy (NA-AION), Visual Field, Optic Nerve Atrophy, Optic Nerve Inflammation

## Abstract

We present a case of Non-Arteritic Anterior Ischemic Optic Neuropathy (NA-AION) with uncertain etiology but a good recovery with a total gain of central visual acuity.

## Introduction

Ischemic optic neuropathy is an acute ischemic disorder of the optic nerve.

It is the most common acute optic neuropathy in patients over 50 years old, with an estimated annual incidence in the United States of 2.3–10.2 per 100 000 population [**1**,**2**].

Ischemic optic neuropathies are of two types: Posterior Ischemic Optic Neuropathy, caused by ischemia of the posterior segment of the optic nerve, vascularized by multiple vessels, and Anterior Ischemic Optic Neuropathy, caused by ischemia of the anterior part of the optic nerve, supplied by the short posterior ciliary artery circulation. Etiologically and pathogenetically, AION is of the following two types: arteritic AION (A-AION), which is due to giant cell arteritis (Horton disease), and nonarteritic AION (NA-AION), which is due to all other causes [**3**,**4**].

NAION presents with loss of vision occurring over hours to days, often described as blurring, dimness, or cloudiness in the affected region of the visual field, most often inferiorly. NAION typically presents without pain, although some form of periocular discomfort is reported in 8–12% of affected individuals [**5**,**6**]. 

Visual acuity in patients with NA-AION varies considerably, from 20/ 20 (logMAR 0) to no perception of light; however, in general, the drop in acuity in patients with NA-AION is less than that experienced by patients with the arteritic form of AION, with over 50% of the patients having acuity better than 20/ 200 (logMAR 1) [**7**]. Color vision loss in NA-AION tends to parallel visual acuity loss, as opposed to that in optic neuritis, in which color loss is often disproportionately greater than visual acuity loss. Visual field defects in NA-AION may follow any pattern related to optic nerve damage, but altitudinal loss, usually inferior, occurs in the majority, ranging from 55 to 80% of the reported cases [**7**-**9**].

## Case report

A 52-year-old Caucasian man was admitted in another ophthalmological service for sudden decrease of visual acuity in the left eye after a brief period of physical effort associated with an event with emotional impact. The eye examination was inconclusive and the patient presented the next day in our clinic. General symptoms were absent at admission.

The patient had no relevant family history or ophthalmological afflictions but he declared a history of anxiety, depression and increased arterial blood pressure. 

At presentation, his best-corrected visual acuity was 20/ 20 (0 logMAR) for the right eye and 20/ 200 (1 logMAR) for the left eye with a small spherical hyperopic correction. The intraocular pressure by applanation tonometry was 17 mmHg in the right eye and 12 mmHg in the left eye.

The findings on external examination and slit-lamp examination of the anterior segment were within normal limits aside from a relative afferent pupillary defect in the left eye.

**Fig. 1:**
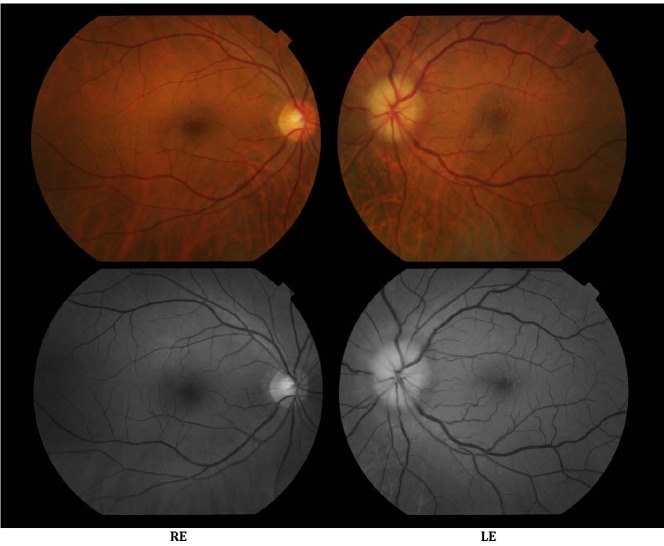
Color and red free fundus photography of the right and left eye

**Fig. 2:**
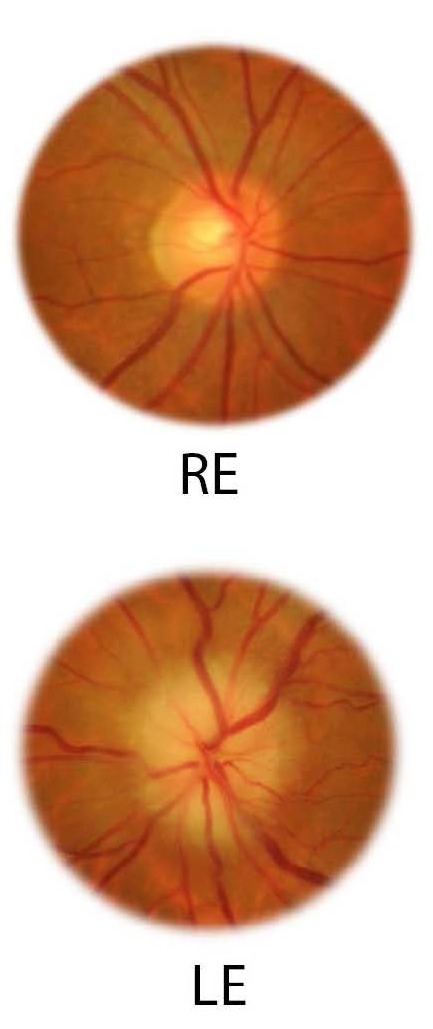
Optic disc detail of the right and left eye

The fundus of each eye was examined after pharmaceutical mydriasis with 0.5% tropicamide and 10% phenylephrine hydrochloride ophthalmic solutions (**Fig. 1**,**2**). The optic nerve disc in the left eye was imprecisely delimited, had a swollen appearance and the cupping was absent, this aspect being highly suggestive for papillary edema. The retinal arteries were narrowed, the veins were turgescent, and the vessels had a concentric arrangement. The macula appeared within normal limits. The ophthalmoscopy examination of the right eye showed no relevant changes.

**Fig. 3:**
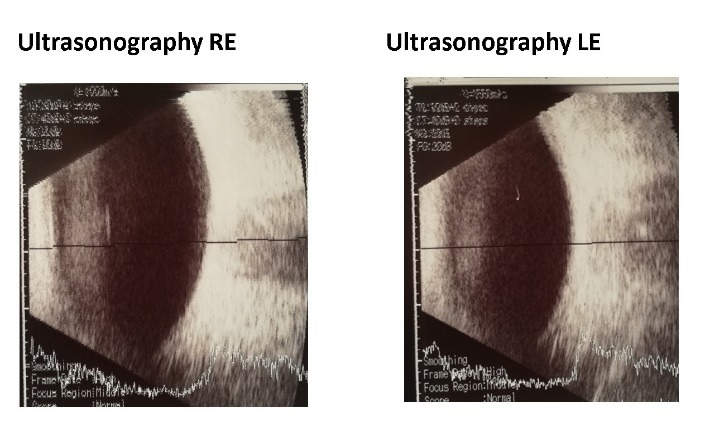
Ultrasonography of the right and left eye

The ultrasonography for the left eye showed a widening (right red arrow) of the hypoechogenity representing the optic nerve sheath, which confirmed the optic nerve edema. The right eye appeared to have no pathological changes (**Fig. 3**).

**Fig. 4:**
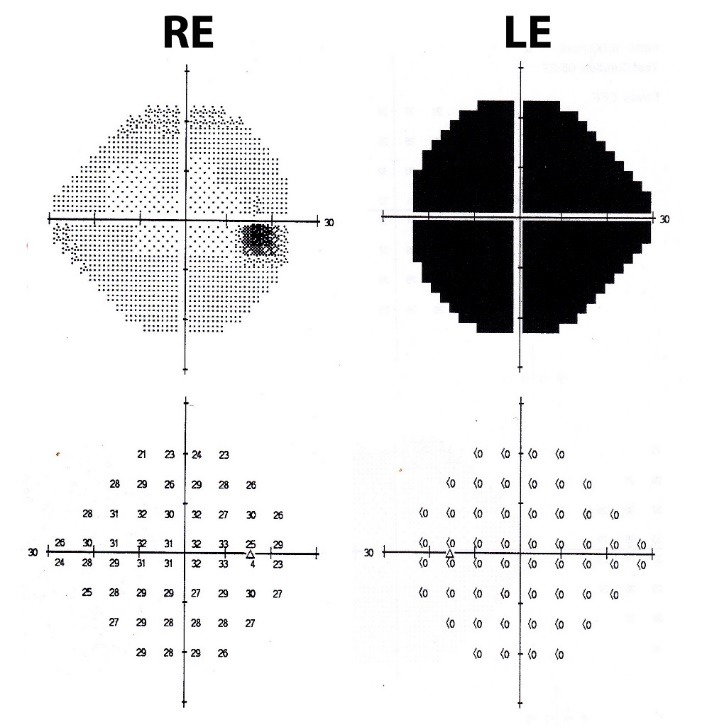
Threshold values maps and grayscale maps from Humphrey visual field of both eyes

Perimetry was assessed by a Humphrey Visual Field Analyzer, central 24-2 threshold program, with a size III white stimulus. Reliability indices were very good in visual fields from both eyes. It demonstrated absolute scotoma in all quadrants of the left eye and it was normal for the right eye (**Fig. 4**). 

**Fig. 5:**
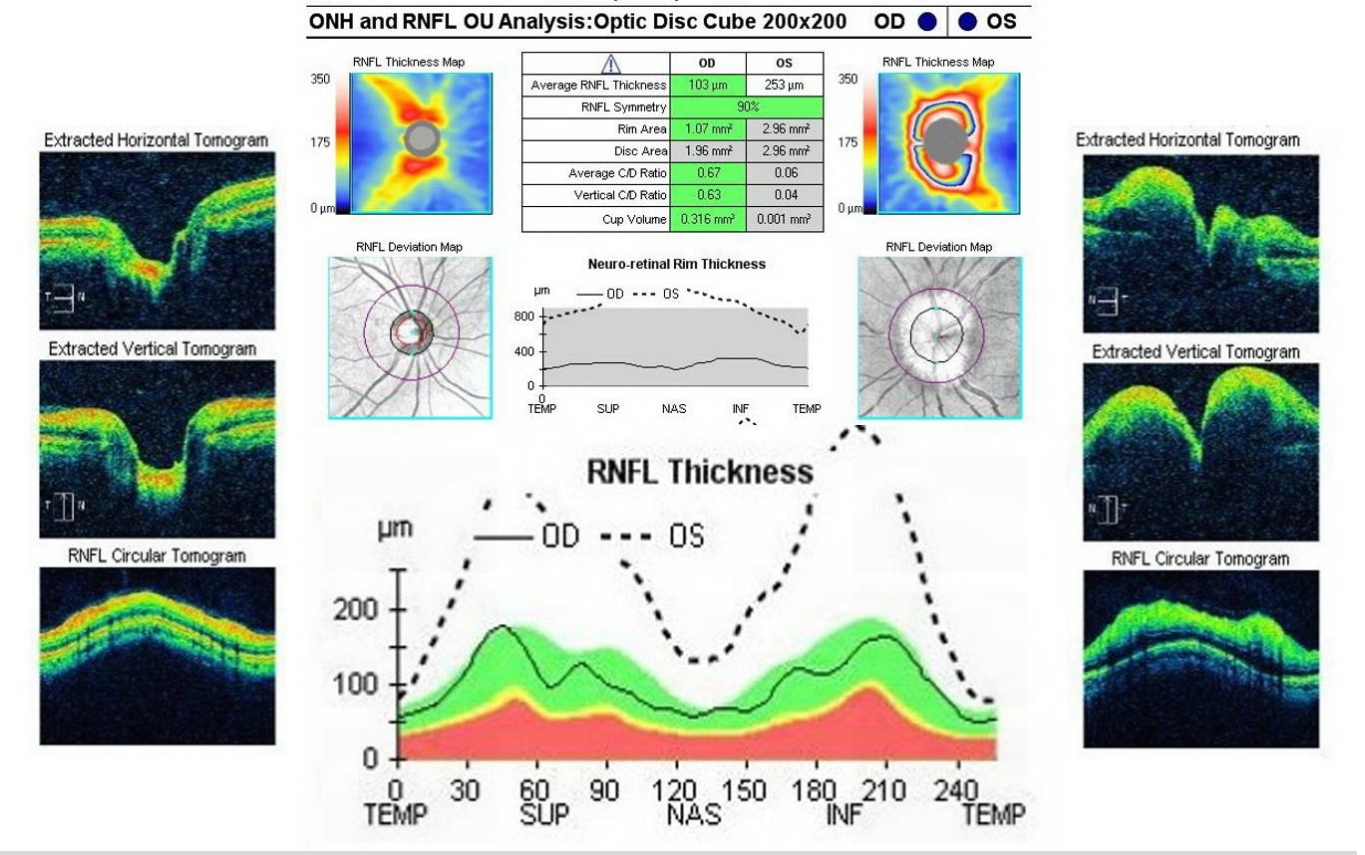
Optical coherence tomography of the optic nerve in both eyes

**Fig. 6:**
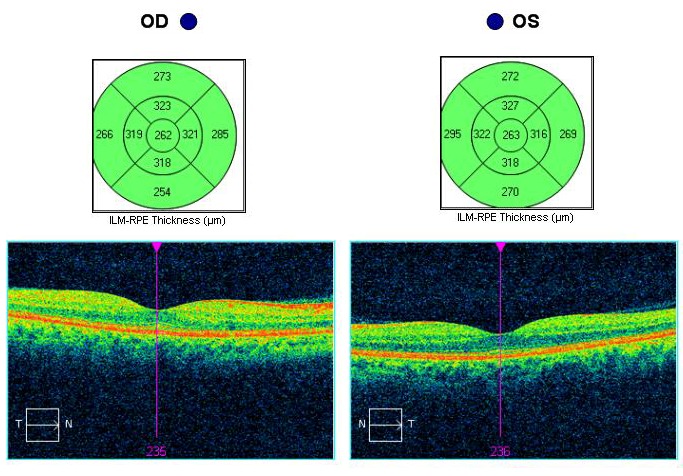
Optical coherence tomography shows normal macular thickness in both eyes

**Fig. 7:**
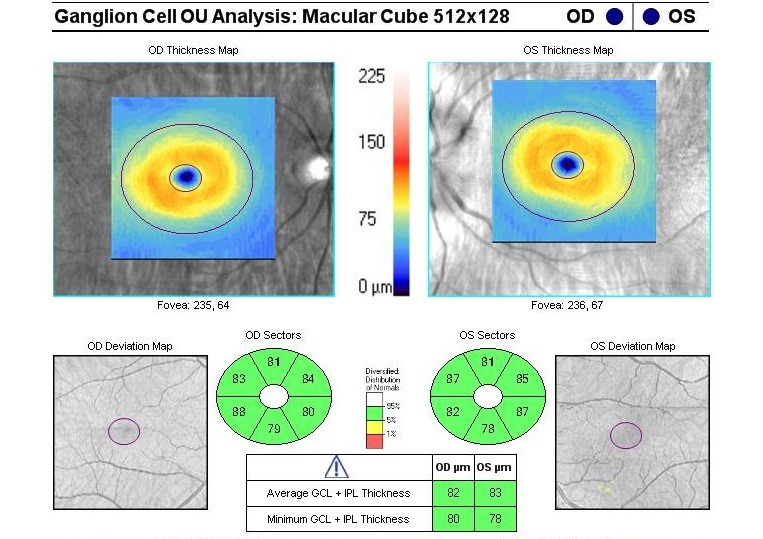
Ganglion cell layer

Optical coherence tomography (OCT) of the optic nerve showed a pseudo thickening of the nerve fiber layer of the left eye (**Fig. 5**). Both the macula and the ganglion cell layer analysis revealed no pathological changes in both eyes (**Fig. 6**,**7**).

Based on this clinical and paraclinical investigations we established the working diagnosis of Papillary Edema of the left eye. The patient was further investigated in order to establish the etiological diagnosis and the course of treatment. 

We further recommended a series of clinical, paraclinical and laboratory complementary investigations. The complete blood count and erythrocyte sedimentation rate had normal values; the biochemistry showed a moderate dyslipidemia and the VDRL was negative. There were no significant findings at the neurological exam, that included a cerebral MRI, and the ENT exam showed the existence of a septum deviation irrelevant to the patient’s ophthalmologic pathology. The endocrinological examination was within normal limits. However, the cardiologic and the dental exam revealed three possible precipitant factors: medically controlled stage II arterial hypertension, atheromatosis, and ipsilateral chronic apical periodontitis.

Considering these complementary investigations, we established the diagnosis of Non-Arteritic Anterior Ischemic Optic Neuropathy of the left eye.

The differential diagnosis included causes of papillary pseudo-edema, as well as papillary edema [**10**-**12**].

**Table 1:** 

Papillary pseudo-edema		Exclusion factors
Congenital anomalies	Oblique papilla	The papilla had a normal orientation.
	Hypermetropic disc	The patient had a small hyperopia of +1,5 sfd.
Optic Nerve Drusen		The ultrasonography showed no ovoid echogenic lesion was present at the junction of the retina and the optic nerve.

**Table 2:** 

Papillary edema		Exclusion factors
Hereditary	Leber Optic Neuropathy	No family history; is usually bilateral and begins in a patient’s teens or twenties.
Traumatic Optic Neuropathy		No traumatic event in the past.
Toxic (CO2) / metabolic (uremia)/ nutritional deficiency		Normal laboratory results.
Intracranial Hypertension	Tumor	Normal head MRI.
	Cerebral venous thrombosis	Normal head MRI.
	Idiopathic (pseudotumor cerebri)	Normal neurological examination.
Compressive	Meningioma	Normal head MRI.
	Thyroid Ophthalmopathy	No infiltration of the retro-orbital space and an endocrinological examination within normal limits.
Infiltrative	Leukemia	Normal laboratory results.
	Sarcoidosis	No history of uveitis, no neovascularization, and chorioretinal lesions, no other systems, and organs affected.
Inflammatory	Infections	Ipsilateral chronic apical periodontitis but with normal inflammatory tests.
	Sarcoidosis	No history of uveitis, no neovascularization, and chorioretinal lesions, no other systems, and organs affected.
	Demyelinating Optic Neuritis	No headache or pain on eye movement; no photopsia and a normal MRI.
Vascular	Central Retinal Vein Occlusion	No retinal hemorrhages, no macular edema or cotton wool spots.
	Malignant hypertension	Drug controlled arterial hypertension.
	AION	Normal inflammatory tests and absence of pain in the temporal region.
	NA-AION	

Given the above exclusion criteria and the fact that the patient presented with several elements common for NA-AION, our positive diagnosis was confirmed. The patient was male, aged between 40 and 60, with acute, monocular, painless and non-progressive visual acuity and visual field loss, relative afferent pupillary defect and papillary edema with spontaneous remission after 8 weeks [**10**,**13**]. It was commonly associated with hyperlipemia and atherosclerosis, both of them being present in this patient.

The patient was followed-up for 10 months. He received vasodilator therapy (Nicergolin 30mg/ day), antithrombotic (Acetylsalicylic acid 75mg/ day) and neuroprotector treatment and we recommended the treatment of the cardiologic and oral pathologies.

The right eye presented with no pathological changes during the follow-up period.

**Fig. 8:**
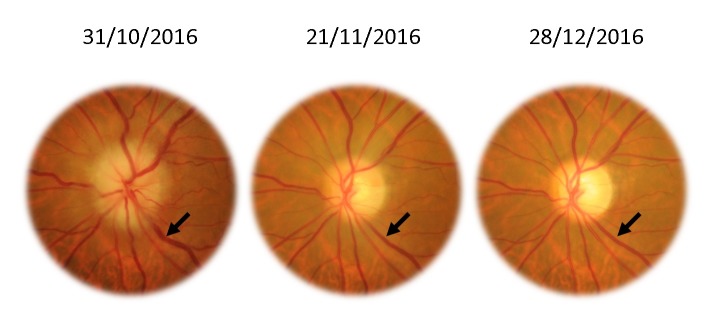
Left eye - remission of the papillary edema and vein turgescence

For the left eye, the best corrected central visual acuity increased from 20/ 200 (1 logMAR) (in October 2016) to 20/ 20 (0 logMAR) (in December 2016). The aspect of the optic disc improved with the remission of the edema in 3 weeks, but, unfortunately, with occurrence of pallor of the disc at 2 months of follow-up (**Fig. 8**). The caliber of the veins also showed an improvement (black arrows).

**Fig. 9:**
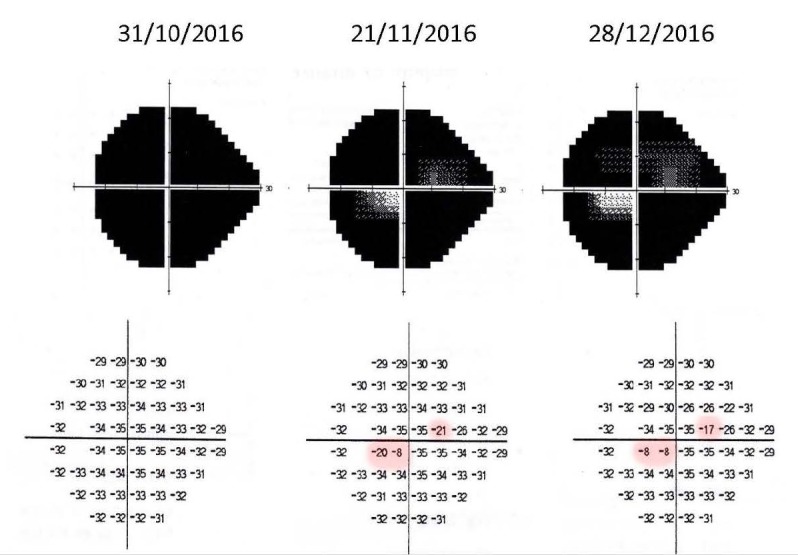
Perimetry evolution in 3 months

Perimetry was assessed at every visit and during the follow-up period there was a mild improvement of the visual field, with the improvement of the central island of vision (**Fig. 9**).

**Fig. 10:**
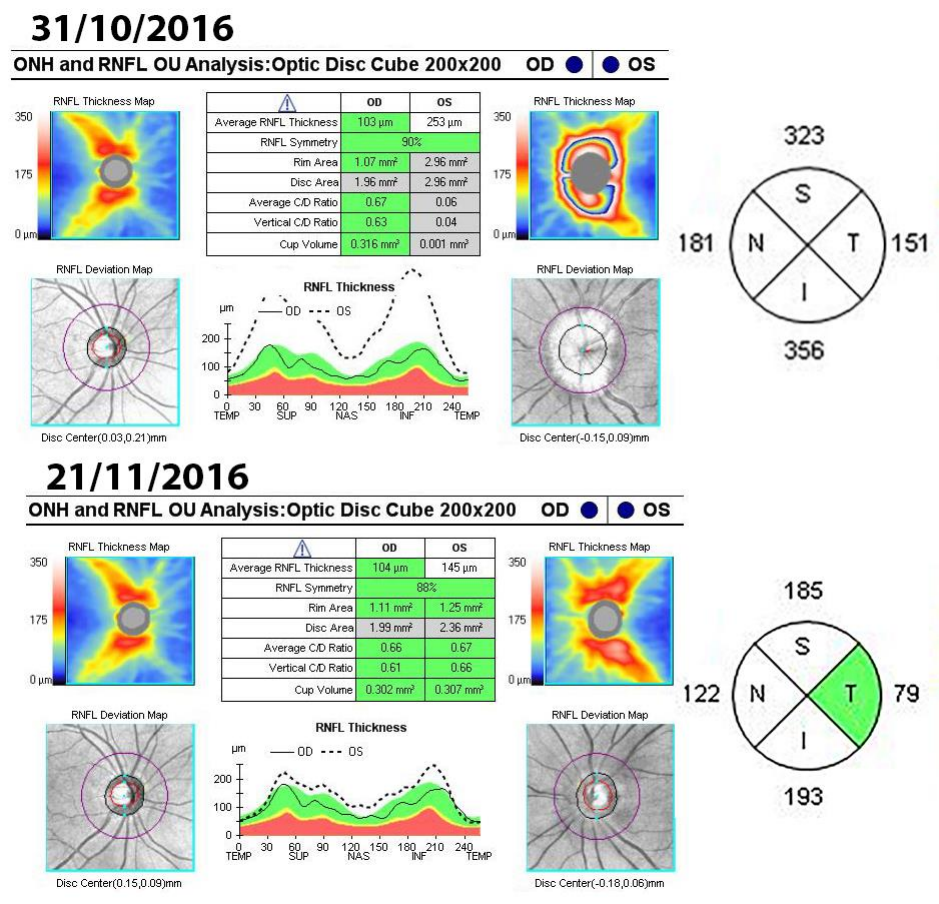
Optic nerve OCT – the apparent average thickness of the RNFL for the left eye decreased form 253µm in October to 142µm in November

The evolution of the optical nerve OCT for the left eye showed an initial regression of the papillary edema at 3 weeks (**Fig. 10**), followed at 2 months by the thinning of the retinal nervous fiber layer. At 6 months, there was a diffuse aspect of the lesions, expanding circumferentially without affecting the nasal quadrant.

**Fig. 11:**
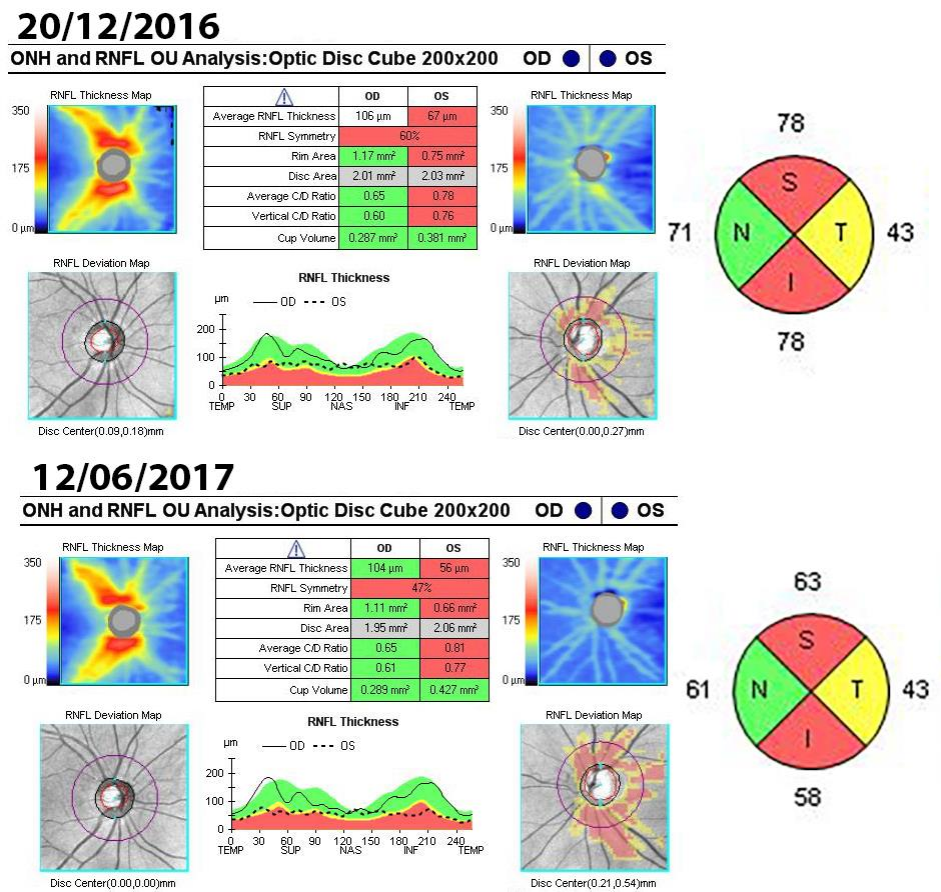
Optic nerve OCT – the apparent average thickness of the RNFL for the left eye decreased even further form 67µm in December to 56µm in June

**Fig. 12:**
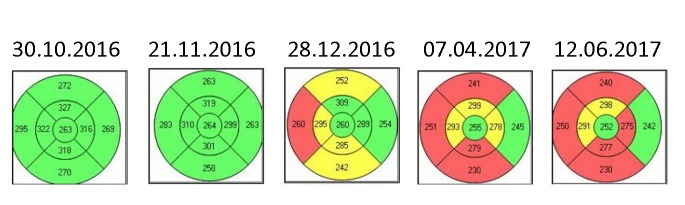
Left eye - Macular OCT evolution from October 2016 to June 2017

**Fig. 13:**
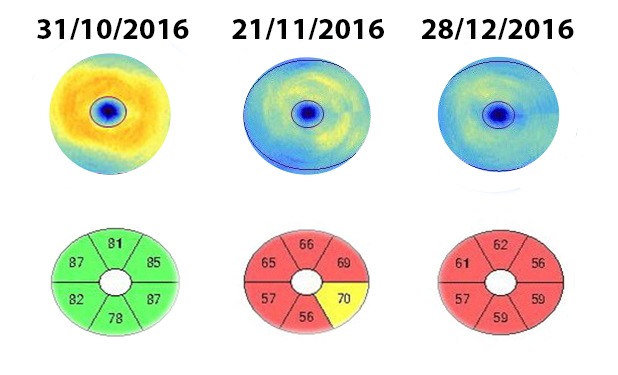
Left eye - Ganglion cell layer atrophy

The macular thickness suffered a progressive decrease, especially in the periphery: upper quadrant from 272µ to 240µ, lower quadrant from 270µ and nasal quadrant from 295µ to 250µ. The central macular thickness decreased from 263µ to 252µ (**Fig. 12**). The macular changes occurred because of the atrophy that gradually appeared in the ganglion cell layer (**Fig. 13**).

The best corrected central visual acuity improved progressively in 3 weeks from 20/ 200 (1 logMAR) to 20/ 20. The visual field had undergone minor changes with the gain of a central island of sight. The optic nerve and ganglion cell atrophy was identified by OCT at 2 months after the beginning of the disease, evolved insidiously for 6 months, and then it stabilized. 

## Discussion

The prognosis of this case is reserved. Optic nerve atrophy is irreversible, and currently there is no effective treatment to help nerve regeneration [**12**,**14**].

There are several theories to be discussed regarding the physiopathology of NA-AION. 

Blood flow through the short posterior ciliary arteries (SPCAs) is reduced in patients with NAION [**15**], however, the rare cases that have been studied histopathologically have shown no evidence of thrombosis of the SPCAs [**16**,**17**] and it is therefore believed that the condition is, in some cases, due to generalized hypoperfusion and in others to occlusion of disc or laminar capillaries. The lack of consistent choroidal filling delay in fluorescein angiography studies of NA-AION suggests that the impaired perfusion arises in the paraoptic tributaries of the SPCA’s, distal to their split from the choroidal branches [**18**].

Persistent hypoperfusion requires impairment in the normal autoregulatory mechanisms of the optic nerve head. Flow is normally maintained constant with variations in perfusion pressure, intraocular pressure, and metabolic conditions (including tissue oxygen and CO2 levels) by factors that vary resistance to flow, such as the autonomic input. These autoregulatory mechanisms may be impaired by arteriosclerosis, chronic arterial hypertension, vasospasm, or medications, including beta-blockers and other antihypertensive medications [**19**].

Structurally small, “crowded” optic discs are associated with NA-AION [**20**], but the mechanism by which this contributes to ischemia has not been completely elucidated, and the role of additional factors such as nocturnal hypotension and sleep apnoea is unproven [**21**-**27**].

Almost all patients who develop NA-AION have a “disc-at-risk” [**20**]. The structure of the optic disc as a relatively inflexible region encompassing the axons of the optic nerve has been implicated in two histopathologic studies suggesting a compartment syndrome as a mechanism [**28**,**29**]. 

Almost all patients who develop NA-AION have at least one underlying vascular risk factor that may or may not be known at the time they lose vision. Nocturnal systemic hypotension may have a significant role in the development of NA-AION, particularly in patients with an exaggerated nocturnal “dip” or in patients, such as those with systemic hypertension, in whom optic disc circulation autoregulatory mechanisms are impaired [**30**].

NAION has been reported in association with many conditions that may predispose to decreased optic nerve head perfusion via microvascular occlusion: systemic hypertension, diabetes mellitus [**31**-**33**], hyperlipidaemia [**34**,**35**]. Smoking is also a significant risk factor on the basis that smokers developed NA-AION at a significantly younger age than nonsmokers [**36**]. 

Several medications have been associated with the development of NA-AION including Interferon-alpha via systemic hypotension or immune complex deposition within the optic disc circulation. Three drugs that inhibit phosphodiesterase 5—sildenafil (Viagra), tadalafil (Cialis), and vardenafil (Levitra) may produce systemic hypotension. Amiodarone is in widespread use as a cardiac anti-arrhythmic agent and has been associated with the development of an anterior optic neuropathy that clinically mimics NA-AION [**37**-**40**].

It is not known whether ischaemia results from local arteriosclerosis with or without thrombosis, embolization from a remote source, generalized hypoperfusion, vasospasm, failure of autoregulation, or some combination of these processes.

Concerning the cellular mechanisms of the NA-AION, recent advances in the understanding of ischaemic central nervous system damage have raised new questions regarding the pathogenesis of neuronal damage in both the arteritic and nonarteritic forms of AION. Neurotrophin deprivation in retinal ganglion cells after ischaemic insult may have a significant role in cell death [**41**,**42**]. Secondary neuronal degeneration in cells adjacent to infarcted tissue may develop as a result of a toxic environment produced by the dying cells [**43**]. Such deterioration may be mediated by processes including excitatory amino acid (especially glutamate) toxicity, reactive oxygen species (including lipid peroxidation), intracellular calcium influx, and apoptosis [**15**,**44**]. Ischaemia-induced cell death may result in release of glutamate, with further cell damage and death by excitotoxic induction of apoptosis [**44**,**45**]. Unique animal models for optic nerve ischaemia bring fluorescein angiography based and electrophysiological evidence of early optic nerve axon damage and delayed retinal ganglion cell loss [**46**].

Interestingly, histological and immunohistochemical assessments, indicate a significant inflammatory reaction in the region of the ischaemic lesion, suggesting that at least some of the optic nerve damage is related to inflammation rather than ischaemia [**47**,**48**].

Several therapeutic approaches have been tried over the years and we are trying to review and comment them.

Mega-dose intravenous corticosteroid treatment was also proposed as a treatment for NA-AION [**49**]. The presumed mechanism through which corticosteroids improve the outcome in NA-AION patients is prevention of the “vicious circle” [**50**] in which the ischemic tissue further suffers from the secondary damage by a mechanical pressure caused by the swollen ischemic axons in an already crowded disc with a small scleral canal. This would not prevent the primary insult but should theoretically limit the secondary insult. Reducing capillary permeability in the optic disc [**51**] could be another mechanism.

It is feasible that if the optic nerve could be “saved” from the secondary damage caused by inflammation [**52**], and the mechanical damage caused by the swelling itself, a boost of IV corticosteroids (as opposed to oral treatment) would be more efficient, as was found for optic neuritis in the ONTT [**53**].

In the Optic Neuritis Treatment Trial three groups of patients with acute optic neuritis received: oral prednisone (1 mg per kilogram of body weight per day) for 14 days; intravenous methylprednisolone (1 g per day) for 3 days, followed by oral prednisone (1 mg per kilogram per day) for 11 days; or oral placebo for 14 days. Visual function was assessed over a six-month follow-up period. Intravenous methylprednisolone followed by oral prednisone speeds the recovery of visual loss due to optic neuritis and results in slightly better vision at six months. Oral prednisone alone is an ineffective treatment and increases the risk of new episodes of optic neuritis.

IV corticosteroids proved to neither improve the final VA nor the final VF of NA-AION patients compared to untreated patients, but only help reach these values faster than with no treatment at all. Of further concern is the list of systemic side effects of corticosteroids and significance of diabetes instability, hypertensive crisis, weight gain and mood instability.

Systemic oral corticosteroid administration showed some evidence of improving visual function compared to the natural evolution of acute NA-AION [**54**]. Other studies revealed no difference in visual outcome between treated and untreated subjects [**55**]. 

Erythropoietin has been shown to be neuroprotective in vitro and in animal models of neuronal injury [**56**], and can be administered intravenously, intravitreally [**57**], or even topically. 

Hyperbaric oxygen was proven to have no beneficial effect [**58**].

The IONDT (Ischemic Optic Neuropathy Decompression Trial) assessed the value of optic nerve sheath fenestration (ONSF) in the treatment of acute NA-AION, based on the theory that at least some of the optic nerve damage caused by NA-AION in which there is progressive visual loss is due to post-ischaemic intraneural swelling with secondary impairment of local vascular flow or axoplasmic transport within the optic nerve head and that reduction of perineural subarachnoid cerebrospinal fluid pressure could improve vascular, axonal transport, or both, thus reducing tissue injury in reversibly damaged axons. However, data analysis revealed no significant benefit of this type of treatment [**59**,**60**].

Transvitreal optic neurotomy has been proposed as a therapy. If a compartment syndrome is at least a component of the pathophysiology of NA-AION, in theory, such a procedure could break the cycle of edema and vascular compression [**61**]. However, this is a rather invasive approach. 

Other studies explored the role of aspirin [**5**,**13**,**30**], vasodilators [**6**], heparin-induced extracorporeal LDL/ fibrinogen precipitation (HELP) [**8**], hyperbaric oxygen [**9**], diphenylhydantoin [**62**], norepinephrine [**63**], levodopa [**64**], topical brimonidine [**65**,**66**] and intravitreal bevacizumab [**20**].

Just as there is no proven treatment for NA-AION, there is no proven prophylaxis to prevent second-eye involvement. Although some authors have found evidence that aspirin can reduce the incidence of fellow-eye involvement after NA-AION [**67**,**68**], others [**69**] found no long-term benefit for aspirin use. Nevertheless, although beneficial long-term effects remain unproven for NA-AION, many experts recommend the use of aspirin after an initial episode, only for its role in decreasing risk for stroke and myocardial infarction in this vasculopathy population group.

NA-AION has a probability of only 5% to relapse in the same eye. There is a risk of congener eye damage: 25% at 3 years, 17% at 5 years and 15% at over 5 years [**10**,**8**].

Particular for this case was that the patient arrived in our clinic at the onset of the symptomatology, and there was a rapid central visual acuity gain, 20/ 20 at 3 weeks, even though there is no efficient treatment demonstrated for NA-AION in the specialty literature. 

The exact cause of the papillary edema has yet to be determined, with no history of hypovolemia, with a drug controlled arterial hypertension and a clinical and paraclinical examination of other systems and organs within normal limits. This causes frustration for both the doctor and the patient.

However, the physical effort and the event with emotional impact, associated with anxiety and arterial hypertension, may have contributed to a hypertensive jump that precipitated the start of NA-AION.

The burden of the treating physician is the chase “towards the end of the tunnel” for the widening of the central area of sight with limited therapeutic resources.

**Financial Disclosures**

None of the other authors has any financial or proprietary interests to disclose.
